# 

**Published:** 1925-01

**Authors:** 


### THE BRISTOL

### Medico-Chirurgical Journal.

A JOURNAL OF THE MEDICAL SCIENCES FOR THE WEST OF ENGLAND AND SOUTH WALES.

*PUBLISHED UNDER THE AUSPICES OF*

*THE BRISTOL MEDICO-CHIRURGICAL SOCIETY.*

EDITOR:

PATRICK WATSON-WILLIAMS, M.D.

WITH WHOM ARE ASSOCIATED

J. LACY FIRTH, M.S., M.D., CAREY COOMBS, M.D.,
E. W. HEY GROVES, M.S., F.R.C.S., B.Sc.

J. A. NIXON, C.M.G., M.D., ASSISTANT EDITOR

EDITORIAL SECRETARY:

A. L. FLEMMING, M.B., B.Ch.

---

"Scire est nescire, nisi id me

Scire alius scierit."

---

*VOL. XLII.*

Library of Congress
548816
JAN 21 1926
Smithsonian Deposit

BRISTOL: J. W. ARROWSMITH LTD.
LONDON: J. W. ARROWSMITH (LONDON) LTD.
1925.

### CONTENTS OF VOLUME XLII.

*Page*

Postgraduate Medical Education. By J. Odery Symes, M.D., D.P.H. 1

Appendicitis as a Cause of Intestinal Obstruction. By Richard Warren, M.D., M.Ch. Oxon., F.R.C.S. .. .. .. .. 23

A Comparative Review of Spinal and Peripheral Nerve Injuries. By Wilfrid Adams, M.S., F.R.C.S. .. .. .. .. 32

University of Bristol: Royal Opening of the New Buildings. By J. A. Nixon, C.M.G., M.D. Cantab. .. .. .. .. 73

A Note on Asthma. By J. R. Charles, M.D., F.R.C.P. .. 84

Diagnosis of the Dyspepsias. By A. Rendle Short, M.D., B.S., B.Sc., F.R.C.S. .. .. .. .. .. .. .. 88

The Present Position of Surgery of the Nervous System. By Percy Sargent, C.M.G., D.S.O., F.R.C.S. .. .. .. .. 101

A Case of Intrathecal Extramedullary Spinal Tumour. By A. Wilfrid Adams, M.S., F.R.C.S. .. .. .. .. .. 116

Some Points in the Differential Diagnosis of Focal Infection in the Nasal Sinuses causing Ocular and other Systemic Infections. By Patrick Watson-Williams, M.D. .. .. .. .. 121

The Diagnosis of Scarlet Fever. By John Wallace, O.B.E., M.B., C.M.Ed. .. .. .. .. .. .. .. .. 157

The Comparative Efficiency of Local Anæsthetics. By Eric Watson-Williams, M.C., Ch.M., F.R.C.S.E. .. .. .. .. 164

Continuity and Change. By T. Carwardine, M.S., F.R.C.S. .. 201

Some Problems in Pulmonary Disease, with Special Reference to Radiography. By H. H. Carleton, M.A., M.D. Oxon., M.R.C.P. 211

The Practical Importance of Posture in the Physical Examination of the Heart, with some Remarks on Stethoscopes. By W. Gordon, M.D., F.R.C.P. .. .. .. .. .. .. .. 224

Reviews of Books .. .. .. .. .. 43, 127, 172, 232

Editorial Notes .. .. .. .. .. .. 53, 138, 188, 241

Meetings of Societies .. .. .. .. .. .. 55, 142

Obituary .. .. .. .. 61, 65, 66, 145, 149, 151, 192, 197

The Library of the Bristol Medico-Chirurgical Society 69, 152, 198, 244

Local Medical Notes .. .. .. .. .. 71, 154, 246

WAC 10/28/42

*Just published. Crown 8vo. 400 pp.* **12s. 6d.** *net. Postage* **6d.** *With 21 Original plates of photomicrographs and other illustrations.*

### RHEUMATIC HEART DISEASE

**By CAREY F. COOMBS, M.D., F.R.C.P. (Lond.)**

With an Introduction by F. J. POYNTON, M.D., F.R.C.P. (Lond.)

"A most important and able contribution to the subject. It is eminently practical and adapted equally for the general practitioner and for the specialist."—*Brit. Med. Jour.*

---

*Recently published. Demy 8vo, 244 pp. With 17 plates (one fully coloured) and other illustrations.* **12s.** *net. Postage* **6d.**

### EPIDEMIC ENCEPHALITIS

**(Encephalitis Lethargica).**

**By ARTHUR J. HALL, M.A., M.D. (Camb.), F.R.C.P. (Lond.),**

*Prof. of Medicine, Univ. of Sheffield; Senr. Phys. Sheffield Royal Hospital.*

"A comprehensive treatise on epidemic encephalitis which neither neurologist nor general physician can afford to exclude from his bookshelf."—*Lancet.*

---

*Demy 8vo, 424 pp. With 222 Text Illustrations and 12 plates, of which 8 are in colour.* **20s.** *net. Postage* **9d.**

### DISEASES OF THE NOSE, THROAT AND EAR

**For Practitioners and Students.**

**Edited by A. LOGAN TURNER, M.D., F.R.C.S. (Ed.),**

*Senior Lecturer on Diseases of the Ear, Nose, and Throat, University of Edinburgh.*

With the Collaboration of

**J. S. FRASER, M.B., F.R.C.S. (Ed.)** **J. D. LITHGOW, M.B., F.R.C S. (Ed.)**
**W. T. GARDINER, M.C., M.B., F.R.C.S. (Ed.)** **G. EWART MARTIN, M.B., F.R.C.S. (Ed.).**

*Lecturers on Diseases of the Ear, Nose, and Throat, University of Edinburgh.*

and

**DOUGLAS GUTHRIE, M.D., F.R.C.S. (Ed.)**

*Lecturer on Diseases of the Ear, Nose and Throat, School of Medicine of the Royal Colleges, Edinburgh.*

---

*Crown 8vo. 138 pp. Stiff Covers, with blank interleaves for Notes.* **5/-** *net. Postage* **3d.**

### A COURSE OF PRACTICAL PHYSIOLOGY.

**Introductory to Physiology and Medicine.**

**By G. A. BUCKMASTER, B.A., D.M. (Oxon),** *Professor of Physiology, Univ. of Bristol,*
**and H. R. B. HICKMAN, M.A., M.B., B.Ch. (Oxon),** *Demonstrator of Physiology, Univ. of Bristol.*

"A most admirable little handbook for the laboratory."—*Physiological Abstracts.*

---

*Fifth Edition. Fully Revised. Crown 8vo. 338 pp. Coloured Frontispiece.* **9s. 6d.** *net. Postage* **6d.**

### THE NEW PHYSIOLOGY IN SURGICAL AND GENERAL PRACTICE.

**By A. RENDLE SHORT, M.D., B.S., B.Sc.Lond., F.R.C.S. Eng.,**

*Exam. in Physiol. for first F.R.C.S.; Surg., Bristol Roy. Infirm.; Lect. on Physiol., Univ. of Bristol.*

"May be cordially recommended to all readers wishing to keep abreast of recent progress in physiology."—*Brit. Med. Journ.*

---

**BRISTOL, ENG.: JOHN WRIGHT & SONS LTD.**

**LONDON: SIMPKIN, MARSHALL, HAMILTON, KENT & CO. LTD.**

### Bristol Medico-Chirurgical Society.

---

### President.

J. ODERY SYMES, M.D. Lond.

### President-Elect.

T. CARWARDINE, M.S. Lond.

### Committee.

*Ex-Officio*
- J. LACY FIRTH, M.S. Lond. (Retiring President).
- P. WATSON-WILLIAMS, M.D. Lond. (Editor of *Journal*).
- C. K. RUDGE (Hon. Librarian).
- A. L. FLEMMING, M.B., Ch.B. Brist. (Editorial Secretary).

*Elected.*

F. LACE } Ex-Presidents.
J. A. NIXON, C.M.G., B.A., M.D. Cantab. }
D. C. RAYNER.
C. FERRIER WALTERS.
E. W. HEY GROVES, M.D., M.S. Lond.
A. J. WRIGHT, M.B., B.S. Lond.
H. L. ORMEROD, M.D. (R.U.I.).
W. VINCENT WOOD, M.C., B.A. Cantab.

### Honorary Secretary.

S. V. STOCK, M.B., B.S. Lond., M.B., B.Ch. Brist.

Medical Practitioners wishing to join the Society are requested to communicate with the Hon. Secretary, Mr. S. V. STOCK, 1 Mortimer Road, Clifton. The Annual Subscription is £1 11s. 6d.

---

*Extracts from Journal By-laws.*

4.—The *Journal* shall be delivered free to Subscribers and also to Members of the Society whose subscriptions are not in arrear.

6.—The Annual Subscription to the *Journal* is 10/6.

---

Communications intended for insertion in the *Journal* should be sent to the Editor, Dr. P. WATSON-WILLIAMS, 2 Rodney Place, Clifton, Bristol.

Books for Review and Exchange Journals should be sent to the Assistant-Editor, *Bristol Medico-Chirurgical Journal*, Medical Library, The University, Bristol.

Communications referring to the delivery of the *Journal* should be sent to the Editorial Secretary, A. L. Flemming, 48 Pembroke Road, Clifton, Bristol, to whom Subscriptions are to be paid *in advance*.

Cases for Binding Vols. I—XLI are now ready, and may be obtained, price 1/9 each (postage extra), upon application to J. W. ARROWSMITH LTD., Quay Street, Bristol; or the *Journal* will be bound, including Case, for 7/- per volume.

### Bristol Medico-Chirurgical Society.

### PAST PRESIDENTS.

1874—75. FREDERICK BRITTAN, M.D., B.A. Dub.

1875—76. ROBERT WILLIAM COE.

1876—77. SAMUEL MARTYN, M.D. Ed.

1877—78. AUGUSTIN PRICHARD, M.D. Berlin.

1878—79. HENRY EDWARD FRIPP, M.D. Würzburg.

1879—80. WILLIAM MICHELL CLARKE.

1880—81. JOSEPH GRIFFITHS SWAYNE, M.D. Lond.

1881—82. EDWARD LONG FOX, M.D. Oxon.

1882—83. JAMES GEORGE DAVEY, M.D. St. And.

1883—84. WILLIAM JOHNSTONE FYFFE, M.D., B.A. Dub.

1884—85. GEORGE FORSTER BURDER, M.D. Aberd.

1885—86. WILLIAM HENRY SPENCER, M.D., M.A. Cantab.

1886—87. FRANCIS POOLE LANSDOWN.

1887—88. LEMUEL MATTHEWS GRIFFITHS.

1888—89. ROBERT SHINGLETON SMITH, M.D., B.Sc. Lond.

1889—90. NELSON CONGREVE DOBSON, Ch.M. Bristol.

1890—91. SAMUEL HENRY SWAYNE.

1891—92. FRANCIS RICHARDSON CROSS, M.B.Lond., LL D.Bristol.

1892—93. EDWARD MARKHAM SKERRITT, M.D., B.S., B.A. Lond.

1893—94. JAMES GREIG SMITH, M.B., C.M., M.A. Aberd., F.R.S.E.

1894—95. ALFRED JAMES HARRISON, M.B. Lond.

1895—96. ARTHUR WILLIAM PRICHARD.

1896—97. ALFRED EDWARD AUST LAWRENCE, M.D., C.M. Aberd.

1897—98. JOHN EDWARD SHAW, M.B., C.M. Ed.

1898—99. ROBERT ROXBURGH, M.B. Ed.

1899—00. WILLIAM HENRY HARSANT.

1900—01. DAVID SAMUEL DAVIES, M.D., Lond.

1901—02. BARCLAY JOSIAH BARON, M.B., C.M. Ed.

1902—03. GEORGE MUNRO SMITH, M.D. Bristol.

1903—04. JAMES PAUL BUSH, C.M.G., C.B.E., Ch.M. Bristol.

1904—05. REGINALD EAGER, M.D. Lond.

1905—06. JOHN DACRE.

1906—07. JAMES TAYLOR.

1907—08. HENRY WALDO, M.D., C.M. Aberd.

1908—09. JOHN MICHELL CLARKE, M.D., M.A. Cantab.

1909—10. JAMES SWAIN, C.B., C.B.E., M.D., M.S. Lond.

1910—11. BERTRAM MITFORD HERON ROGERS, M.D., B.Ch., B.A. Oxon.

1911—12. CHARLES ALEXANDER MORTON.

1912—13. WALTER CARLESS SWAYNE, M.D., B.S. Lond. and Bristol.

1913—14. PATRICK WATSON-WILLIAMS, M.D. Lond.

1914—15. WILLIAM HARRY CHRISTOPHER NEWNHAM, M.A., M B. Cantab.

1915—16 GEORGE PARKER, M.A., M.D. Cantab.

1916—17 FRANCIS HENRY EDGEWORTH, M.A., M.D. Cantab., D.Sc. Lond.

1917—18 FREDERICK LACE.

1918—19 ROBERT GUTHRIE POOLE LANSDOWN, M.D., B.S. Durh.

1919—20 I. WALKER HALL, M.D., Ch.B. Vict.

1920—21 LEOPOLD ERNEST VALENTINE EVERY-CLAYTON, M.D. Lond.

1921—22 CYRIL HUTCHINSON WALKER, M.B., B.A. Cantab.

1922—23 JOHN ALEXANDER NIXON, C.M.G., B.A., M.D. Cantab.

1923—24 JOHN LACY FIRTH, M.D., M.S. Lond.

1925.

---

LIST OF SUBSCRIBERS

TO THE

### Bristol Medico-Chirurgical Journal.

---

### HONORARY MEMBERS OF THE SOCIETY.

| | |
|---|---|
| Owen, Sir Isambard, D.C.L., M.D. | Abbey Gate, Beach Road, Weston-super-Mare. |
| Rudge, C. King ... ... ... ... ... | 145 Whiteladies Rd., Clifton |

---

### SUBSCRIBERS.

*Those marked * are Members of the Society.*

| | |
|---|---|
| *Ackland, W. R. ... ... ... ... ... ... ... | 5 Rodney Place, Clifton. |
| *Adams, A. W., M.B., B.Sc. Lond. ... ... ... | 9 Mortimer Road, Clifton. |
| Adye, W. J. A. ... ... ... .. ... ... ... | St. Margarets, Bradford-onAvon, Wilts. |
| *Alexander, A., M.A. ... ... ... ... ... .. | 23 Manilla Road, Clifton. |
| *Alexander, D. A., M.B., Ch.B. Vict. ... ... | 30 Berkeley Square, Clifton. |
| *Askins, R. A., M.D., B.Ch., B.A.O. Dubl.... | Guildhall, Bristol. |
| *Aubrey, T., M.B. Lond. ... ... ... ... ... | Bitton, near Bristol. |
| *Baker, Lily A., M.D., B.Ch., B.A.O., R.U.I.... | 21 Victoria Square, Clifton. |
| *Barber, M. C., M.B., Ch.B. Brist. ... ... ... | Ingledene, High Street, Staple Hill, Bristol. |
| *Barker, G. H., M.D., B.Sc. Lond. ... ... ... | 124 Redland Road, Bristol. |
| *Bartholomew, E. U. ... ... ... . ... ... | 12 Lansdown Place, Clifton. |

### LIST OF SUBSCRIBERS.

*Bergin, F. G. ... ... ... ... ... ... ... ... ... 31 Oakfield Road, Clifton.

Bernard, C. ... ... ... ... ... ... ... ... ... 564 Fishponds Road, Fishponds, Bristol.

Berry, F. C., M.D., B.Ch., B.A. Dub. ... ... Locarno, Burnham, Somerset.

*Birrell, J. A., M.B., B.S. Lond. ... ... 51 Sussex Place, Ashley Road.

Blackett, J. F., M.D., B.S. Lond. ... ... ... Hamsterley, Bloomfield Park, Bath.

*Blagg, Arthur F., M.D. Durh.... ... ... ... 6 Upper Belgrave Road, Clifton.

*Blathwayt, A. de V.... ... ... ... ... ... 6 Brock Street, Bath.

Bletchly, G. Playne, M.B. ... .. ... ... Hazelwood, Nailsworth, Glos.

*Bodman, C. O., M.D. Durh. ... ... ... ... 17 Upper Belgrave Road, Clifton.

*Bodman, J. Hervey, M.D. Lond. ... ... ... 9 Whiteladies Road, Clifton.

*Bowker, G. E., M.D. Edin.... ... ... ... ... 11 North Parade, Bath.

*Bradbeer, E. G. ... ... ... ... ... ... ... ... 32 Linden Road, Westbury Park.

*Bray, G., Lt.-Col., *D.S.O.* ... ... ... ... 26 Beaconsfield Road.

*Brentnall, T. C. ... ... ... ... ... ... ... ... Mendip Hills Sanatorium, Wells.

*Bristowe, H. C., M.D. Lond. ... ... ... ... Wrington, Somerset.

*Buckmaster, G. A., M.A., M.D. Oxon. ... ... University of Bristol.

*Burdon-Cooper, J., M.D., B.Sc. Durh. ... ... 12 The Circus, Bath.

*Bush, J. Paul, *C.M.G.*, *C.B.E.*, Ch.M. Brist. Vyvyan House, Clifton.

*Cairns, F. J. ... ... ... ... ... ... ... ... ... Devon House, Whitehall Road, Bristol.

*Carey, R. S., *O.B.E.*, B.A. Cantab. ... ... 502 Bath Road, Brislington, Bristol.

*Carleton, H. H., M.A., M.D. B.Ch. Oxon. 1 Lansdown Road, Clifton.

*Carling, A., M.A., M.B., B.C. Cantab. ... ... 12 Great George Street, Bristol.

*Carwardine, Thomas, M.B., M.S. Lond. ... 16 Victoria Square, Clifton.

*Cave, Edward J., M.D. Lond. ... ... ... ... 20 The Circus, Bath.

*Chambers, E. R. ... ... ... ... ... ... ... 9 Clifton Park, Clifton.

*Charles, J. R., M.A., M.D., B.C. Cantab. ... Deepholm, Clifton.

*Chitty, H., M.S. Lond. ... ... ... ... ... ... 46 Pembroke Road, Clifton.

*Christofferson, P. E., M.B., Ch.B. Brist. ... 8 Lansdown Place, Clifton.

Claremont, L. E. ... ... ... ... ... ... ... 5 Rodney Place, Clifton.

*Clarke, R. C., *O.B.E.*, M.B., Ch.B. Brist. ... 29 Victoria Square, Clifton.

*Coates, Vincent M., *M.C.*, M.A., M.D B.C. Cantab. .. ... ... ... ... ... ... ... 10 The Circus, Bath.

Connellan, P. S. ... ... ... ... ... ... ... ... 12 Bloomsbury Street, London, W.C.1.

*Coombs, Carey, M.D. Lond. ... ... ... ... 3 Pembroke Road, Clifton.

*Cooper, William Russell ... ... ... ... 13 Westfield Park, Redland, Bristol.

*Corfield, C., M.D. Durh. ... ... ... ... 5 Kensington Place, Clifton.

Cossham, W. R., M.D., C.M. Aberd.... ... ... Wellesley House, Cirencester.

Cridland, A. B. ... ... ... ... ... ... ... Salisbury House, Chapel Ash, Wolverhampton.

*Cross, F. R., M.B. Lond., LL.D. Brist. ... Worcester House, Clifton.

*Crossman, F. W., M.B. Durh. ... ... ... ... White's Hill, Hambrook near Bristol.

*Crouch, C. P. ... ... ... ... ... ... ... ... 5 Harley Place, Clifton.

*Dacre, John ... ... ... ... ... ... ... ... 14 Eaton Crescent, Clifton.

*Datta, S., M.B., B.Ch. Bris. ... ... ... ... Stapleton Institution, Bristol.

*Davies, E. D. D. ... ... ... ... ... ... ... Standish House, Stonehouse, Glos.

*Davies, D. S., M.D. Lond., D.P.H. Cantab. ... 6 Lansdown Place, Clifton.

### LIST OF SUBSCRIBERS.

| | |
|---|---|
| DEVINE, H., M.D Lond. | Corporation Mental Hospital, Portsmouth. |
| *DEVIS, H. F. | Shrubbery House, Shrubbery Road, Downend. |
| *DIX, C. | 11 Victoria Square, Clifton. |
| *DIXON, G. C., B.A., M.B. | Bristol Eye Hospital. |
| *DIXON, T. B. | 134 Coronation Road, Bristol. |
| *DRAPES, T. L., M.B., B.C., B.A. Cantab. | St. Maur, Beaufort Square, Chepstow. |
| *DRIVER, H. LLOYD | 6 Victoria Square, Clifton. |
| DYMOKE, F. | 21 Cotham Road, Bristol. |
| *EDGEWORTH, F. H., M.D., B.C., M.A. Cantab., D.Sc. Lond. | 13 Richmond Hill, Clifton. |
| EDWARDS, C. D., B.A., M.D. Cantab. | The Corderies, Chalford, Glos. |
| *EGLINGTON, J. H. C. | Street, Somerset. |
| *ELKINGTON, G. W., M.B., B.S. Lond. | Bruton, Somerset. |
| ELLIOT BROS. LTD. | *Australian Pharmaceutical Notes & News* O'Connell Street, Sydney, N.S.W. |
| ELLIOTT, F. PERCY, M.B., C.M. Aberd. | 113 Grove Rd., Walthamstow, London, E.17. |
| *ELLIOTT, W. H. A., M.B., B.S. Lond. | 2 Oakfield Road, Clifton. |
| *EVERY-CLAYTON, L. E. V., M.D. Lond | The Old School House, Tetbury. |
| *FAILL, C. J. C. | 19 Portland Square, Bristol. |
| FARNFIELD, W. W. | Clive Vale, Gillingham, Dorset. |
| *FALLON, Col. J. | 35 Cavendish Road, Henleaze, Bristol. |
| *FAWN, G. F. | 6 Oakfield Road, Clifton. |
| *FELLS, A., M.B., C.M. Edin. | 6 Elton Road, Clifton. |
| *FIRTH, J. L., M.D., M.S. Lond. | 8 Victoria Square, Clifton. |
| *FLEMMING, A. L., M.B., Ch.B. Brist. | 48 Pembroke Road, Clifton. |
| *FLEMMING C. E. S. | Manvers House, Bradford-on-Avon. |
| *FOSS, E. V., M.D., Ch.B. Brist. | Clouds Hill House, St. George, Bristol. |
| *FRASER, A. D., M.A., M.B., Ch.B. | 4 Alexandra Road, Clifton. |
| *GARDEN, R. R., M.A., M.B., B.Ch. | 9 Harley Place, Clifton. |
| GARLAND, E. B., M.B., C.M. Edin. | 114*a* Hampton Road, Redland, Bristol. |
| *GEE, C. A. H., M.B., Ch.B. Brist. | Portbury, Nr. Bristol. |
| *GERRISH, D. S. | 328 & 330 Church Road, St. George, Bristol. |
| *GIBBS, A. N. GODBY | 52 Whiteladies Road, Clifton. |
| *GLENNY, E. T., M.B., B.S. Lond. | 102 Pembroke Road, Clifton |
| *GOLDING, MADGE E., M.B., Ch.B. | 76 Cranbrook Road, Redland. |
| *GORDON, R. G. M.D., B.Sc. Edin. | 9 Circus, Bath. |
| *GREEN, T. A., *D.S.O.*, M.D., C.M. Edin. | 33*a* Whiteladies Road, Clifton. |
| GRIFFITHS, CORNELIUS A. | 35 Newport Road, Cardiff. |
| GRIFFITHS, J. S. | 20 Redland Park, Bristol. |
| *GRIFFITHS, S. J. H. | Bristol General Hospital. |
| *GROVES, E. W. HEY., M.D., M.S. Lond. | 25 Victoria Square, Clifton. |

*Hadfield, G., M.D. Lond. ... ... ... ... General Hospital, Bristol.

*Hailes Clements D. G., M.D., C.M. Ed. ... 2 Richmond Park Road, Clifton.

*Hall, E. George, M.B. Lond. ... ... ... ... 42 Apsley Road, Clifton, Bristol.

*Hall, I. Walker, M.D., Ch.B. Vict. ... ... Manilla Lodge, Clifton.

*Harris, H. Elwin, B.A., M.B. Cantab. ... ... 13 Lansdown Place, Clifton.

*Harsant, W. H. ... ... ... ... ... ... ... ... Tower House, Clifton Down Road, Clifton.

*Harty, J. P. I., M.B., B.Ch., B.A.O., R.U.I. Deepholm, Clifton.

*Hawkins, E. J. ... ... ... ... ... ... ... Constantine House, Avonmouth.

*Herapath, C. E. K., *M.C.*, M.D. Lond. ... ... Ormlie, Clifton.

Hill, Hedley, M.D. Durh. ... ... ... ... 101 Pembroke Road, Clifton.

Hospital, Bristol General (*Secretary*, T. W. Gregg).

Hughes, D. ... ... ... ... ... ... ... ... 12 Cotham Road, Bristol.

*Iles, A. E., *O.B.E.*, M.B., B.S. Lond. ... ... 17 Victoria Square, Clifton.

Infirmary, Bristol Royal (*Secretary*, E. C. Smith).

Ingle, C. Durban ... ... ... ... ... ... ... Somerton, Somerset.

*Jackman, W. A. ... ... ... ... ... ... ... Mortimer Road, Clifton.

*Johnson, R. G., M.B., D.P.H. ... ... ... ... 119 Redland Road, Bristol.

*Joll, C. A., M.B., M.S. Lond. ... ... ... ... 64 Harley Street.

*Jones, J. Ellington ... ... ... ... .. ... 20 Pembroke Road, Clifton.

*Kennedy, W. P. M.D. Dubl. ... ... ... ... 3 Gay Street, Bath.

*Kerby, T. R. R. ... ... ... ... ... ... ... Heywood, Pill, Nr. Bristol.

*Kerfoot, Stanley J. ... ... ... ... ... ... 109 East Street, Bedminster, Bristol.

*Kingston, S. H., M.B., Ch.B. Brist. ... ... 3 Whiteladies Road, Clifton.

*Kyle, H. G., M.A., M.D. B.Ch. Oxon. ... ... 31 Westbury Road, Bristol.

*Lace, F. ... ... ... ... ... ... ... ... ... 5 Gay Street, Bath.

*Lavington, C C., M.B., B.S. Durh. .. ... 1 Burlington Road, Redland, Bristol.

*Lees, E. Leonard, M.D., C.M. Ed. ... ... ... 22 Richmond Hill, Clifton

*Legat, R. Eddowes M.B., C.M. Edin.... ... Murray House Staple Hill, Bristol.

Leigh, W. W. ... ... ... ... ... ... ... ... Treharris, Glamorganshire.

*Leonard, R. C., M.D. Durham ... ... ... ... Bower Ashton, Bristol

*Levis, J. S., *M.C.*, M.B., B.Ch., N.U.I. .... 20 Gay Street, Bath.

| | |
|---|---|
| *LEWIS, D. J. ... ... .. ... ... ... ... ... | 11 Great Bedford Street, Bath. |
| *LINTON, MARION S., M.B. Lond. ... ... ... | 21 Oakfield Road, Clifton. |
| *LISTER, A. E. J., Lt.-Col., I.M.S. (Retired) ... | 12 All Saints Road, Clifton. |
| *LISTER, L. MARGARET ... ... ... ... ... | 12 All Saints Road, Clifton. |
| *LOGAN, H. B. ... ... ... ... ... ... ... ... | Eastfield, Southville, Bristol. |
| *LUCAS, J. J. S., M.D. Lond. ... ... ... ... ... | 186 Wells Road, Totterdown, Bristol. |

| | |
|---|---|
| *MACDONALD, P. T. T., M.A., M.B., Ch.B. ... | 43 Regent Street, Kingswood. |
| MACKINNON, CHARLES, M.B., C.M., M.A. Glasg. | Davaar, Cirencester. |
| MACLEAN, EWEN J., M.D., C.M. Ed.... ... ... | 12 Park Place, Cardiff. |
| *MACLEOD, DONALD, M.B., C.M. Ed. ... ... | 84 Redland Road, Redland, Bristol. |
| *MACLEOD, G., *M.C.*, M.D., Ch.B. ... ... ... | "Wargrave," Clevedon. |
| *MACWATTERS, J. C., *M.B.E.* ... ... ... ... | Almondsbury, near Bristol. |
| *MCKENZIE, W. G., *M.C.* ... ... ... ... ... | 101 Coronation Road, Bristol. |
| *MCLANNAHAN, J. G. ... ... ... ... .. ... | The Mount, Stonehouse. |
| MARSHALL, ARTHUR L., M.B., B.A. Cantab. ... | Thornleigh, Upper Clapton, London, E.5 |
| *MATHER, J. S., M.B., C.M. Aberd. ... ... ... | Lawrence Hill House, Bristol. |
| *MATHEW, W. E., M.D. ... ... ... ... ... ... | 85 Coldharbour Road, Redland. |
| *MAYES, F. J. A. ... ... ... ... ... ... ... ... | 79 Pembroke Road, Clifton. |
| MIALL, C. L'O. ... ... ... ... ... ... ... | Elm Hayes, Paulton, near Bristol. |
| *MILLING, T., M.B., Ch.B. ... ... .. ... ... | 1 Vicarage Road, Southville. |
| *MILNER, A. N. P. ... ... .. ... ... ... ... | 318 Stapleton Road, Bristol. |
| *MOORE, C. A., M.B., M.S. Lond. ... ... ... | 56 St. Paul's Road, Clifton. |
| *MORRIS, A. G. ... ... ... ... ... ... ... ... | 18 Westbury Park, Westbury-on-Trym. |
| MORRIS, L. N., M.B., Ch.B. Brist. ... ... ... | 24 All Hallows Road, Upper Easton, near Bristol. |
| *MORRIS, MARY E. H., M.D. Lond. ... ... ... | 19 Gay Street, Bath. |
| *MORTON, C. A. ... ... ... ... ... ... ... ... ... | 14 Vyvyan Terrace, Clifton. |
| *MUMFORD, W. G., M.B. Lond. ... ... ... ... | 18 The Circus, Bath. |
| *MYLES, G T. ... ... ... ... ... ... ... ... | 7 Apsley Road, Clifton. |

| | |
|---|---|
| *NEILD, NEWMAN, M.B., B.Ch. Vict. ... ... ... | 9 Richmond Hill, Clifton. |
| *NEWNHAM, W. H. C., M.B., M.A. Cantab. ... | 3 Lansdown Place, Victoria Square, Clifton. |
| *NICHOLS, F. C., *M.C.*, M.B., Ch.B. Brist. ... | 38 Whiteladies Road, Clifton. |
| *NIXON, H. C., M.D. Edinb. ... ... ... ... | 5 Queen's Parade, Bath. |
| *NIXON, J. A., *C.M.G.*, M.D. Cantab. ... ... | 7 Lansdown Place, Clifton. |
| *NOTT, WINIFRED, M.B., Ch.B., Brist. ... ... | "Fassiefern," Upper Cranbrook Road. |

| | |
|---|---|
| OPPENHEIMER, SON & CO. LTD. ... ... ... | 179 Queen Victoria Street, London, E.C.4. |
| *ORMEROD, H. LAWRENCE, M.D., B.Ch. R.U.I. | 2 Henleaze Road, Westbury-on-Trym, Bristol. |
| ORR-EWING, H. J. *M.C.* M.D. Lond. ... ... | English Mission Hospital, Jerusalem. |

| | |
|---|---|
| *Parker, George, M.D., M.A. Cantab. ... ... | 14 Pembroke Road, Clifton. |
| *Parkes, J. A., M.B., Ch.B. Liverpool. ... ... | 2 Effingham Road, Bristol. |
| Parkinson, Major G. S., *D.S.O.*, R.A.M.C.... | R.A.M.C. Mess, Grosvenor Place, London, S.W. 1. |
| *Parsons, H. F. ... ... ... ... ... ... ... ... | The Heath, Hazelwood Road, Sneyd Park, |
| Parsons, Sir J. H., M.B., B.S., D.Sc. Lond.... | 54 Queen Anne Street, London, W. |
| Paterson, Donald Rose, M.D., C.M. Ed. ... | 15 St. Andrew's Crescent, Cardiff. |
| *Phillips, J. R. P., *C.B.E.* ... ... ... ... ... ... | Northwoods, Winterbourne. |
| *Pollard, J., M.B., B.Ch., B.A.O. ... ... ... | 52 Cumberland Road, Bristol. |
| Powell, J. J., M.D. Lond. ... ... ... ... ... ... | 2 Gloucester Road, Bishopston, Bristol. |
| Price, D. T. M.B. Lond. ... ... ... ... ... ... | Castle Cary, Somerset. |
| *Pridie, H. H., M.B., C.M. Ed. ... ... ... | 17 Oakfield Road, Clifton. |

| | |
|---|---|
| *Rayner, D. C. ... ... ... ... ... ... ... ... | 9 Lansdown Place, Clifton, Bristol. |
| *Renton, R. A. S., *M.C.*, M.D. Durh.... ... ... | Tortworth House, Clevedon. |
| *Robertson, D., M.B. Ch.B. Edin. ... ... ... | 205 Wells Road, Knowle. |
| *Rogers, Beatrice, ... ... ... ... .. .. ... | Richmond Hill, Clifton. |
| *Rogers, B. M. H., M.D., B.Ch., B.A. Oxon. ... | 14 Mortimer Road, Clifton. |
| *Rolfe, R., B.A., M.B., B.C. Cantab. ... ... | Park House, St. Andrews Road, Avonmouth. |
| *Rose, F. H. ... ... ... ... ... ... ... ... | 8 Chantry Road, Clifton. |
| *Rutherford, J. M., M.B., C.M. Edin. ... ... | Brislington House, near Bristol. |

| | |
|---|---|
| *Salisbury, Walter, M.D., M.S. Lond. ... | Glanford, Oswold Road, Scunthorpe, Lincs. |
| Savage, W. G., M.D. ... ... ... ... ... ... ... | "Bourn," 7 Trewartha Park, Weston-super-Mare. |
| *Shaw J. E., M.B., C.M., Edinb. ... ... ... | 23 Caledonia Place, Clifton. |
| *Short, A. R., M.D., B.S., B.Sc. Lond. ... ... | 69 Pembroke Road, Clifton. |
| *Short, L. J., M.D., B.S. Lond., M.D. Brist. ... | Grove Park House, Weston-super-Mare. |
| *Smith, G. Cockburn, M.D. Brux. ... ... ... | 12 South Road, Newton Abbot. |
| Smith, Rev. Reginald, M.A. Cantab. ... ... | The Vicarage, Walpole St. Andrew, Norfolk. |
| *Smith, W. Alexander M.B., M.A. Oxon. ... | 70 Pembroke Road, Clifton. |
| *Smyth, Richard, M.A., M.D. Dub. ... ... | Burnley, Westbury-on-Trym. |
| *Smythe, H. J. D., *M.C.*, M.B., M.S. ... ... | 72 St. Paul's, Road, Clifton. |
| *Staniland, M. F. ... ... ... ... ... ... ... | 255 Stapleton Road. |
| *Statham, R. S. S., *O.B.E.*, M.D., M.Ch. Brist. | 24 College Road, Clifton. |
| *Stock, W. S. V., M.B., B.S. Lond., M.B., B.Ch. | 1 Mortimer Road, Clifton. |
| *Stewart, A. L., D.M., D.Ch. Brux. ... ... | 31 Howard Road, Southville. |
| *Stuart, Agnes, M.B., Ch.B. ... ... ... ... | Raleigh Road, Bedminster, Bristol. |
| *Swayne, W. Carless, M.D. Lond. ... ... ... | 2 Clifton Park, Clifton. |
| *Symes, J. O., M.D. Lond. ... ... ... ... ... | 71 Pembroke Road, Clifton. |

| | |
|---|---|
| *Tasker, D. G. C., M.B., M.S. Lond. ... ... | 16 All Saints Road, Clifton. |
| Taylor, A. L. ... .. ... .. ... ... ... ... | The Royal Infirmary, Bristol. |
| *Taylor, H. N., M.A. Cantab., M.D. Dub. ... | Hillside, Ebbw Vale. |
| *Taylor, James ... ... ... ... ... ... ... ... | 36 Alma Road, Clifton. |
| *Taylor, J. W., O.B.E., M.D. ... ... ... ... | 8 Redcliffe Parade, Bristol. |
| *Temple, G. H., M.B., C.M. Ed. ... ... ... | Ailanthus, Weston-super-Mare. |
| *Templeton, W. L., M.B., Ch.B. Glasgow ... | 143 Cheltenham Road, Bristol. |
| *Terry, C. H., M.A., M.B., B.Ch. Oxon. .. ... | 2 Gay Street, Bath. |
| Thin, James ... ... ... ... ... ... ... ... ... | 54 & 55 South Bridge, Edinburgh. |
| *Thomas, J. D., M.B. Cantab. ... ... ... ... | Northwoods, Winterbourne. |
| *Todd, A. T., *O.B.E.*, M.B., Ch.B. Edin. ... | Clayton, Clifton Park, Clifton. |
| *Trist, J. R. R., *M.C.* ... ... ... ... ... ... | 120 Henleaze Road, Clifton. |
| *Tryon, V., M.B., Ch.B. Brist. ... ... ... ... | 3 Harley Place, Clifton. |

| | |
|---|---|
| *Vickery, W. H. ... ... ... ... ... ... ... ... | 1 Trewartha Park, Weston-super-Mare. |

| | |
|---|---|
| *Walker, Cyril H., M.B., B.A. Cantab. ... ... | 8 Oakfield Road, Clifton. |
| Walker, H. B. ... ... ... ... ... ... ... ... | 12 The Avenue, Minehead. |
| *Wallace, John, *O.B.E.*, M.B., C.M. Edin. ... | Lauriston, Weston-super-Mare. |
| *Wallace, J. T., M.B., B.Ch., B.A. R.U.I. ... | 48 Coronation Road, Bristol. |
| *Walters, C. F. ... ... ... ... .. ... ... | 5 Mortimer Road, Clifton. |
| *Wanklyn-James, C. W., *O.B.E.* ... ... ... | 3 Mortimer Road, Clifton. |
| *Warren, R., M.A., M.D. Oxon. ... ... ... | 1 Royal Terrace, Weston-super-Mare. |
| *Waterhouse, R., M.D. Lond. .. ... .. ... | 25 Circus, Bath. |
| *Watson-Williams, P., M.D. Lond.... ... ... | 2 Rodney Place, Clifton. |
| *Watson-Williams, E., *M.C.*, M.B., B.Ch., B.A. Cantab. ... ... ... ... ... ... ... | 12 Victoria Square, Clifton. |
| *Whicher, A. H. ... ... ... ... ... ... ... ... | 115 Queen's Road, Clifton. |
| Whitehouse, H. Beckwith, M.S. ... ... | 52 Newhall Street, Birmingham. |
| Wigan, C. A., M.D. Durh., J.P. ... ... ... ... | Deepdene, Portishead. |
| Wigmore, J., M.D. R. U. I. ... ... ... ... ... | 16 Queen Square, Bath. |
| *Wilkinson, S. H., M.D. Edin. ... ... ... | Pensions Hospital, Ashton Court. |
| *Willcox, J. W. J., M.B., B.S. Lond. ... ... | Glastonbury. |
| Willcox, R. Lewis ... ... ... ... ... ... | 97 London Road, Salisbury. |
| *Williams, E. C., M.B., B.C., B.A. Cantab. ... | 159 Whiteladies Road, Clifton. |
| *Williams, G. C., B.A. Cantab. ... ... ... | Town Hall, Oxford. |
| *Williams, L. H., M.D. Durh. ... ... ... ... | Thornbury, Glos. |
| Williams, S. R., M.B. Lond. ... ... ... ... | Buckfastleigh, Devon. |
| *Wills, W. K., M.B., B.C. Cantab. ... ... ... | 19 Whiteladies Road, Clifton. |
| *Wood, Duncan ... ... ... ... ... ... ... ... | 5 Eaton Crescent, Clifton. |
| *Wood, W. V., *M.C.* ... ... ... ... ... ... | Henley Lodge, Yatton, Som. |
| *Wright, A. J., M.B., B.S. Lond. ... ... ... | 14 Victoria Square, Clifton. |

### The Library of the Bristol Medico-Chirurgical Society.

*The following donations have been received since the publication of the List in October,* 1924.

*January*, 1925.

| | |
|---|---|
| American College of Physicians (1) | 1 volume. |
| British Medical Association (2) | 1 ,, |
| Carnegie Institution of Washington (3) | 4 volumes. |
| Delegates of the Clarendon Press (4) | 1 volume. |
| Dr. David Hooper (5) | 2 volumes. |
| London School of Tropical Medicine (6) | 1 volume. |
| Medical Research Council (7) | 1 ,, |
| Dr. G. Parker (8) | 1 ,, |
| Rockefeller Foundation (9) | 1 ,, |
| Dr. Serge Voronoff (10) | 1 ,, |

Unbound periodicals have been received from Dr. Carey Coombs and Mr. Hey Groves.

### THE ONE HUNDRED AND ELEVENTH LIST OF BOOKS.

The figures in round brackets refer to the figures after the names of the donors. The books to which no such figures are attached have either been bought from the Library Fund or received through the *Journal.*

**Benedict, F. G., & Ritzman, E. G.** *Undernutrition in Steers* (3) 1923

**Cameron, S. J.** .. *A Glasgow Manual of Obstetrics* .. .. .. 1924

**Coombs, Carey F.** *Rheumatic Heart Disease* .. .. .. .. 1924

**Clarke, E.** .. .. *Refraction of the Eye* .. .. .. 5th Ed. 1924

**Clayton-Greene, W. H.** [Ed.] *Pye's Surgical Handicraft* 9th Ed. 1924

**Clayton, E. Bellis** .. *Physiotherapy in General Practice* .. .. 1924

**Davenport, C. B.** .. *Body-build and its Inheritance* .. .. (3) 1923

**Dumarest, F. & Brette, P.** *La pratique du Pneumothorax thérapeutique* .. .. .. .. .. .. .. .. .. 1923

**Fox, E. M.** .. .. *First Steps in Nursing* .. .. .. .. .. .. 1924

**Fuller, A. W.** .. *Anæmia: its Causes and Modern Treatment* 2nd Ed. 1924

**Godlee, Sir R. J., Bart.** *Lord Lister* .. .. .. .. .. (4) 3rd Ed. 1924

**Hall, P.** .. .. .. *Ultra-Violet Rays in the Treatment and Cure of Disease* .. .. .. .. .. .. .. .. 1924

*Handbook for Queen's Nurses* .. .. .. .. .. .. .. .. .. 1924

**Hewitt, C. R.** [Ed.] *The Medical Year Book, 1925* .. .. .. 1924

**Humphris, P. H.** .. *Artificial Sunlight and its Therapeutic Uses* .. 1924

**Hurst, A.** [Ed.] .. *Guy's Hospital Reports, Oct., 1924* .. .. .. 1924

**Hutchison and Rainy.** *Clinical Methods* .. .. .. .. .. 8th Ed. 1924

**Joslin, E. P.** .. .. *Diabetic Metabolism with High and Low Diets* .. .. .. .. .. .. .. .. .. (3) 1923

**Leslie, R. Murray** *Pneumonia: its Pathology, Diagnosis, etc.* .. 1924

**May and Worth** .. *Diseases of the Eye* .. .. .. .. .. 5th Ed. 1924

**Miles, W. R.** .. *Alcohol and Human Efficiency* .. .. (3) 1924

**Rawling, L. Bathe** *Landmarks and Surface Markings of the Human Body* .. .. .. .. .. .. .. .. 6th Ed. 1924

**Rolleston, J. D.** .. *Acute Infectious Diseases* .. .. .. .. .. 1925

**Rose and Carless** .. *Manual of Surgery* .. .. .. .. .. 11th Ed. 1924

**Saywell, E.** .. .. *Sidelights from the New Psychology* .. .. 1924

**Semon, Donaldson and Others.** *A Synopsis of Special Subjects* 1924

**Thomson, F. H.** .. *Diagnosis and Treatment of the Infectious Diseases* .. .. .. .. .. .. .. .. 1924

**Thomson, J. G.** .. *Researches on Blackwater Fever in Southern Rhodesia* .. .. .. .. .. .. .. (6) 1924

**Tubby, A. H.** .. *The Advance of Orthopædic Surgery* .. .. 1924

**Voronoff, S.** .. .. *Quarante-trois greffes du Singe à l'Homme* .. 1924

" .. .. *Greffe Animale* .. .. .. .. .. .. .. (10) 1924

**Walker, J. H.** .. *The Modern Nursing of Consumption* 2nd Ed. 1924

TRANSACTIONS, REPORTS, JOURNALS, &c.

American College of Physicians, Year Book, 1923-24 .. .. (1) 1924
American Pediatric Society, Transactions .. Vol. XXXVI. 1924
American Pharmaceutical Association, Year Book, 1920 and 1922 (5)
British Medical Association, Annual Handbook, 1924-25 .. .. (2) 1924
London Dermatological Society, Transactions and 13th Annual Report .. .. .. .. .. .. .. .. .. .. .. .. .. .. 1924
Medical Research Council, Report 89. Miners' 'Beat Knee,' etc. (7) 1924
Proceedings of the Third International Congress of the History of Medicine .. .. .. .. .. .. .. .. .. .. .. .. .. (8) 1923
Rockefeller Foundation, Annual Report, 1923 .. .. .. (9) 1923
St. Andrew's Institute for Clinical Research, Report of Vol. II. 1924
St. Thomas's Hospital Reports .. .. .. .. Vol. 46, 1922 1924
Surgical Clinics of North America .. Vol. 4, No. 4, Aug., 1924 1924

---

### Local Medical Notes.

---

MEDICAL SCHOOL DINNER.

The Annual Dinner was held on December 11th at the Grand Hotel, Bristol. Mr. W. E. Miles, F.R.C.S., was the Guest of the Evening, and M. A. L. Flemming presided over a company numbering some 150. The President proposed the toast of "The King," and Dr. Charles Corfield then proposed the toast of "The Guest of the Evening." He spoke of Mr. Miles' position in the front rank of those surgeons who specialise in the lower alimentary tract, and of his work at the Cancer and Gordon Hospitals. Mr. Miles proposed the toast of the "Bristol Medical School," and referred to its long role of distinguished men. Surgeon-Commander Blatchford, R.N., responded for the Medical School. Professor Buckmaster proposed the toast of "The President," and Mr. Flemming suitably replied. A reception was afterwards given at the University by the wives of the Staff of the Faculty of Medicine.

EXAMINATION RESULTS.

University of Bristol.—Students of the University have recently passed the following examinations :—

M.B., Ch.B. Bristol.—*Part II.* (*Forensic Medicine and Toxicology only*) : R. H. Dummett.

Diploma in Dental Surgery.—*Special Final Examination* : W. B. Bowrey, W. H. Greet, D. Griffiths, C. H. Hill, W. H. Phillips.

M.B. London.—(*With Honours and Distinction in Medicine*): J. A. James, M.B., Ch.B., Bristol.

F.R.C.S. Eng. (Primary).—F. Langford, M.B., Ch.B., Bristol.

F.R.C.S. Edin.—W. Jackman.

L.R.C.P., M.R.C.S.—Doris E. Joscelyne, F. G. Mogg, C. V. Powell.

Conjoint Board.—*Final Examination, Medicine*: Y. M. de la Pasture, H. Rogers. *Surgery*: A. S. Cox, Dorothy E. Crellin, Constance I. Ham, D. C. Prowse, Sylvia M. Tasker-Smith. *Midwifery*: Elizabeth E. Benson, G. W. R. Bishop, Constance I. Ham, J. Hughes, W. G. R. Morris, L. G. Robertson, H. J. Satchwell, F. H. Woolley.

L.S.A.—*Midwifery*: Beryl Davis.

University of Bristol.—Students of the University have recently passed the following Supplementary Preliminary Science Examination for Dental Students, September, 1924:—

*In Physics and Chemistry* (*Completing Examination*): P. J. Whiteman. *In Physics only*: H. G. Clapp. *In Biology only*: C. R. H. Edwards.

B.D.S.—*2nd Examination in Dental Materia Medica* (*Completing Examination*): C. S. Cossham.

L.D.S.—*Final Examination*: G. C. Brooks, A. M. Cawsey, G. C. Friend, H. F. D. Lane, B. F. Robinson.

D.P.H.—J. Ledingham.

The Long Fox Lecture will be delivered in December next by Dr. Carey Coombs. The subject will be announced later.

### APPOINTMENTS.

### *Bristol Royal Infirmary.*

S. H. Kingston, M.B., Ch.B., D.P.H., has been appointed to the new post of Dermatologist.

Eric Watson-Williams, M.C., M.B., M.Ch., F.R.C.S. Ed., has been appointed Assistant Surgeon to the Ear, Nose, and Throat Department.

### *University of Bristol.*

Dr. D. C. Rayner has been appointed Director of Clinical Obstetrics.

### PUBLICATIONS RECEIVED.

From Edward Arnold & Co., London:
*A Glasgow Manual of Obstetrics.* Edited by S. J. Cameron, M.B. (Glas.), F.R.F.P. and S.G. 1924. Price 21s. net.

From Baillière, Tindall & Cox, London:
*The Errors of Accommodation and Refraction of the Eye.* By Ernest Clarke, M.D., F.R.C.S. Fifth Edition. 1924. Price 8s. 6d. net.
*A Manual of Diseases of the Eye.* By Charles H. May, M.D. (New York), and Claude Worth, F.R.C.S. (Eng.). Fifth Edition. 1924. Price 15s. net.
*Manual of Surgery* (Rose and Carless). By Albert Carless, C.B.E., M.B., M.S., F.R.C.S. Eleventh Edition. 1924. Price 20s. net.
*Physiotherapy in General Practice and for the use of Masseuses.* By E. Bellis Clayton, M.B., B.Ch. 1924. Price 10s. 6d. net.

From the British Medical Association:
*Annual Handbook.* 1924-25. Price 2s. 6d. net.

From Cassell & Co. Ltd., London:
*Clinical Methods.* By Robert Hutchison, M.D., F.R.C.P., and Harry Rainy, M.D., F.R.C.P. (Ed.), F.R.S.E. Eighth Edition. 1924. Price 12s. 6d. net.

From William Heinemann Ltd., London:
*Ultra-Violet Rays in the Treatment and Cure of Disease.* By Percy Hall, M.R.C.S., L.R.C.P. With Introduction by Sir Henry Gauvain, M.A., M.D., M.C., and Leonard E. Hill, M.B., F.R.S. 1924. Price 7s. 6d. net.
*Pneumonia: its Pathology, Diagnosis, Prognosis and Treatment.* By the late R. Murray Leslie, M.A., B.Sc., M.D. Edited and revised by J. Browning Alexander, M.D., M.R.C.P. 1924. Price 12s. 6d. net.
*The Medical Year Book,* 1925. Edited by Chas. R. Hewitt. Second Annual Issue. Price 12s. 6d. net.
*Acute Infectious Diseases.* By J. D. Rolleston, M.A., M.D. (Oxon.). 1925. Price 12s. 6d. net.

From H. K. Lewis & Co. Ltd., London:

*The Diagnosis and Treatment of the Infectious Diseases.* By F. H. Thomson, M.B., C.M., D.P.H. 1924. Price 7s. 6d. net.

*Anæmia: its Causes and Modern Treatment.* By A. W. Fuller, M.D. (Edin.). Second Edition. 1924. Price 6s. net.

*Landmarks and Surface Markings of the Human Body.* By L. Bathe Rawling, M.B., B.C., F.R.C.S. Sixth Edition. 1924. Price 7s. 6d. net.

*A Synopsis of Special Subjects.* By H. C. Semon, M.A., M.D., M.R.C.P., and Others. 1924. Price 18s. net.

*The Advance of Orthopædic Surgery.* By A. H. Tubby, C.B., C.M.G., F.R.C.S. Price 7s. 6d. net.

From Humphrey Milford, Oxford University Press:
*Artificial Sunlight and its Therapeutic Uses.* By Francis H. Humphris, M.D., F.R.C.P., M.R.C.S., etc. 1924. Price 8s. 6d. net.
*Reports of the St. Andrew's Institute for Clinical Research.* Vol. II., 1924. Price 10s. 6d. net.

From the Scientific Press Ltd., London:
*First Steps in Nursing.* By E. Margaret Fox, R.R.C., S.R.N. Revised Edition. 1924. Price 3s. net.
*Sidelights from the New Psychology.* By Evelyn Saywell, L.R.C.P., L.R.C.S. (Edin.) 1924. Price 3s. net.
*The Modern Nursing of Consumption.* By Dr. Jane H. Walker. Second Edition. 1924. Price 2s. 6d. net.
*Handbook for Queen's Nurses.* By some Queen's Superintendents. 1924. Price 1s. 6d. net.

From Wakley & Son Ltd., London:
*Guy's Hospital Reports.* October, 1924. Price 12s. 6d. net. Annual Subscription £2 2s. net, post free.

From John Wright & Sons Ltd., Bristol:
*The British Journal of Surgery.* Vol. XII., No. 46. October, 1924. Price 12s. 6d. net. Yearly subscription 42s. net.
*Rheumatic Heart Disease.* By Carey F. Coombs, M.D., F.R.C.P. 1924. Price 12s. 6d. net.
*Pye's Surgical Handicraft.* Edited and largely rewritten by W. H. Clayton-Greene, C.B.E., B.A., M.B., B.C., F.R.C.S. Ninth Edition. 1924. Price 21s. net.

### Bristol Medico-Chirurgical Journal.

Published Quarterly (demy 8vo)
under the auspices of the Bristol
Medico-Chirurgical Society.

---

*An excellent means of advertising*

---

The only Medical Journal published in the West of England, and has an extensive circulation not only amongst the Medical men of Bristol, but also amongst those of the adjoining Counties and South Wales, and in exchange goes to all parts of the world.

Terms for Advertisements:

Whole Page, £2 2s.; Half Page, £1 5s.; Quarter Page, 15s. per issue.

*PRICES FOR SPECIAL POSITIONS ON APPLICATION.*

J. W. ARROWSMITH LTD.,

Advertising Agents,

11 Quay Street,

BRISTOL.

